# Draft Genome Sequence of *Streptomyces* sp. SPMA113, a Prajinamide Producer

**DOI:** 10.1128/genomeA.01126-16

**Published:** 2016-10-13

**Authors:** Hisayuki Komaki, Akira Hosoyama, Natsuko Ichikawa, Watanalai Panbangred, Yasuhiro Igarashi

**Affiliations:** a Biological Resource Center, National Institute of Technology and Evaluation (NBRC), Chiba, Japan; b NBRC, Tokyo, Japan; c Department of Biotechnology and Mahidol University-Osaka University Collaborative Research Center for Bioscience and Biotechnology, Faculty of Science, Mahidol University, Bangkok, Thailand; d Biotechnology Research Center and Department of Biotechnology, Toyama Prefectural University, Toyama, Japan

## Abstract

We report here the draft genome sequence of *Streptomyces* sp. SPMA113 isolated from soil in Thailand. This strain produces a new modified peptide, prajinamide, which has adipocyte differentiation activity. The genome harbors at least 30 gene clusters for synthases of polyketide and nonribosomal peptide, suggesting its potential to produce diverse secondary metabolites.

## GENOME ANNOUNCEMENT

Actinomycetes are recognized as the richest source of a variety of bioactive secondary metabolites. Among this group, members of the genus *Streptomyces* are the most prolific producers of secondary metabolites (1). In our screening for structurally unique secondary metabolites from *Streptomyces*, a new modified peptide, prajinamide, was isolated from the culture broth of a soil-derived actinomycete strain, *Streptomyces* sp. SPMA113, collected in Thailand (2). To assess the potential of the strain to produce other secondary metabolites, including polyketides and nonribosomal peptides, we sequenced the genome of *Streptomyces* sp. SPMA113.

*Streptomyces* sp. SPMA113 is preserved as TP-A0896 and NBRC 110612 at Toyama Prefectural University and the NBRC culture collection, respectively. The whole genome of a *Streptomyces* sp. SPMA113 monoisolate was read by using a combined strategy of shotgun sequencing with GS FLX+ (Roche; 70.2 Mb sequences, 6.1-fold coverage) and paired-end sequencing with HiSeq 1000 (Illumina; 917.6 Mb sequences, 79.1-fold coverage). These reads were assembled using a Newbler version 2.8 software and subsequently finished using the GenoFinisher software (3), which led to a final assembly of 62 scaffolds and eight contig sequences of >500 bp each. The total size of the assembly was 11,592,136 bp, with a G+C content of 71.1%. Coding sequences were predicted by Prodigal (4). To assess biosynthetic potential for polyketide and nonribosomal peptide compounds, polyketide synthase (PKS) and nonribosomal peptide synthetase (NRPS) gene clusters were analyzed in the same manner previously reported (5).

This genome contains at least 11 type I PKS gene clusters, one type II PKS gene cluster, 11 NRPS gene clusters, and seven hybrid PKS/NRPS gene clusters. Type I PKS gene clusters for synthesis of elaiophylin (6) and nigericin (7) are present in Scaffold01. PKS genes for L-155,175 synthesis are encoded in Scaffold03 and Scaffold21, but the gene cluster split into two scaffolds in the draft genome sequence. A galbonolide-synthetic type I PKS gene cluster (8) is present in Scaffold03. An NRRS gene cluster responsible for laspartomycin synthesis (9) is present in scaffold00021. Hybrid PKS/NRPS gene clusters for synthesis of padanamide (10) and hygrocin (11) are present in scaffold00004 and scaffold00011, respectively. Geldanamycin- and meridamycin-synthetic hybrid PKS/NRPS gene clusters (12, 13) also exist in the genome, but these two clusters split into multiple scaffolds (geldanamycin in scaffold00003 and scaffold00022, and meridamycin in scaffold00011, scaffold00029, scaffold00048, and scaffold00052). Since the remaining PKS and NRPS gene clusters, except for these nine stated above, display no significant similarities to the gene clusters whose products are characterized, their products are unable to be predicted at present.

Although this strain is reported to produce prajinamide, geldanamycin, and elaiophylin (2), other secondary metabolites have not been isolated yet. Recently, genome mining is often employed to discover unknown metabolites from *Streptomyces* members (14). Therefore, the genome sequence of *Streptomyces* sp. SPMA113 will provide useful information to further explore uncharacterized secondary metabolites.

### Accession number(s).

The draft genome sequence of *Streptomyces* sp. SPMA113 has been deposited in the DDBJ/ENA/GenBank database under the accession no. BDFA00000000. The version described in this paper is the first version, BDFA01000000.
